# Neuroprotective Effects of Rhynchophylline Against Ischemic Brain Injury via Regulation of the Akt/mTOR and TLRs Signaling Pathways

**DOI:** 10.3390/molecules190811196

**Published:** 2014-07-30

**Authors:** Houcai Huang, Rongling Zhong, Zhi Xia, Jie Song, Liang Feng

**Affiliations:** 1 Animal Center, Jiangsu Provincial Academy of Chinese Medicine, Jiangsu, Nanjing 210028, China; E-Mails: huang-hhc@sina.com (H.H.); wency@163.com (R.Z.); xiazhi0707@126.com (Z.X.); 2 Key Laboratory of Delivery Systems of Chinese Meteria Medica, Jiangsu Provincial Academy of Chinese Medicine, Jiangsu, Nanjing 210028, China; E-Mail: wpaasj@126.com

**Keywords:** rhynchophylline, pMCAO, ischemic stroke, Akt/mTOR, BDNF

## Abstract

Rhynchophylline (Rhy) is an alkaloid isolated from *Uncaria* which has long been recommended for the treatment of central nervous diseases. In our study, the neuroprotective effect of Rhy was investigated in a stroke model, namely permanent middle cerebral artery occlusion (pMCAO). Rats were injected intraperitoneally once daily for four consecutive days before surgery and then received one more injection after surgery. The protein and mRNA levels of p-Akt, p-mTOR, apoptosis-related proteins (p-BAD and cleaved caspase-3), TLR2/4/9, NF-κB, MyD88, BDNF and claudin-5 were examined. Following pMCAO, Rhy treatment not only ameliorated neurological deficits, infarct volume and brain edema, but also increased claudin-5 and BDNF expressions (*p* < 0.05). Moreover, Rhy could activate PI3K/Akt/mTOR signaling while inhibiting TLRs/NF-κB pathway. Wortmannin, a selective PI3K inhibitor, could abolish the neuroprotective effect of Rhy and reverse the increment in p-Akt, p-mTOR and p-BAD levels. In conclusion, we hypothesize that Rhy protected against ischemic damage, probably via regulating the Akt/mTOR pathway.

## 1. Introduction

Stroke is the third leading cause of death in the USA. About 795,000 Americans suffer from stroke annually [1]. Given the complex pathology, therapy for ischemic stroke remains a serious challenge. The neurotoxic side effects greatly confine the application of tissue plasminogen activator, which is the only approved drug on the market, for acute treatment of stoke [2]. New therapies that can target multiple pathways are in greatly need.

Several pathophysiological mechanisms contribute to brain damage induced by cerebral ischemia, such as inflammatory reactions, neuronal apoptosis, blood-brain barrier disruption, and so on [3]. Several reports have demonstrated that excessive inflammatory and immune responses were the pathophysiological basis of damage in the ischemic brain area following cerebral infarction [4]. Phosphoinositide 3-kinases (PI3Ks) and their downstream target Akt belong to a conserved family of signal transduction enzymes, which plays an important part in regulating inflammatory responses and apoptosis [5]. In the initial hours of cerebral ischemia, p-Akt protein level transiently rises in neurons, and this increment is supposed to be a neuroprotective response [6]. p-Akt will activate the downstream proteins such as Bcl-2-associated death protein (BAD), caspases and others. On the other hand, Toll-like receptors (TLRs) also play an crucial role in cerebral ischemia/reperfusion injury via mediating the inflammatory responses [7]. TLRs can also capture ‘danger signals’ released from necrotic cells and take part in initiating the inflammatory response and cell apoptotic process [8,9]. Moreover, PI3K/Akt signaling was involved in the protection against cerebral injury by modulating TLR2 [10].

*Uncaria rhynchophylla* is a traditional Chinese herb that has long been used to treat neural associated disorders in China [11]. Rhynchophylline (Rhy) is an alkaloid from *Uncaria*, and displays impressive neuroprotective action [12,13,14]. Several studies have indicated that Rhy inhibits Ca^2+^ influx and protects cerebellar granule cells from glutamate toxicity [15]. Via regulation of the TLR and neurotrophin signaling pathways, Rhy exhibited anti-convulsive effects in rats with acute seizures. Rhy could reduce the apoptosis induced by dopamine treatment in human teratocarcinoma cell line [16] and effectively block proinflammatory cytokines release in LPS-activated microglial cells [17]. Whether Rhy could exhibit neuroprotection in permanent focal cerebral ischemia (pMCAO) remains unknown. In this study, we first investigated whether Rhy could attenuate ischemic brain damage and then explored the role of PI3K/Akt and TLRs/nuclear factor-κB (NF-κB) signaling pathways underlying Rhy’s effect in cerebral ischemia.

## 2. Results and Discussion

### 2.1. Effect of Rhy on Cerebral Infarction, Neurological Deficit, and Brain Edema

**Figure 1:**
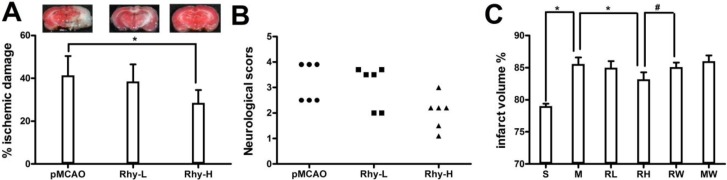
Rhynchophylline attenuated the ischemic damage after pMCAO. (**A**) The bar graph represents the mean ± SE of the ischemic lesion volume. The microphotographs show representative brain slices (* *p* < 0.05 *vs.* pMCAO); (**B**) The scatter graph shows the individual score for neurological deficits; (**C**) The graph shows the percentage of brain edema in Sham (S), pMCAO (M), Rhy-L (RL), Rhy-H (RH), pMCAO + wortmannin (MW) and Rhy-H + wortmannin (RW) groups (* *p* < 0.05 Rhy and Sham *vs.* pMCAO, ^#^ *p* < 0.05 RW *vs.* RH).

We examined the effect of Rhy at two doses, 10 mg/kg and 30 mg/kg, on ischemic brain damage after pMCAO surgery. In the pMCAO group, we noticed a large brain infarction that encompassed the majority of the cortex in the brain hemisphere. The volume of the ischemic brain was considerably reduced in Rhy-H group compared to the control group (41.4% ± 3.7% and 28.5% ± 5.9%, *p* < 0.05) (Figure 1A).

Neurological deficit was examined by a 6-point scale after ischemic stroke (Figure 1B). Relevant neurological deficit appeared in pMCAO group, such as less spontaneous activity and irregular posture. These indicators were remarkably relieved in Rhy-H group but not in Rhy-L group. Sham-operated rats did not show any neurological deficit. Ischemic brain edema was confirmed by assessing the cerebral water content of ischemic brain tissue. The cerebral water content was greatly relieved by Rhy-H treatment, not by Rhy-L treatment (Rhy-H *vs.* pMCAO: 83.2% ± 1.1% *vs.* 85.6% ± 1.0%, *p* < 0.05). Wortmannin, a PI3K inhibitor, abolished the protective effect of Rhy (Figure 1C).

### 2.2. Effects of Rhy on p-Akt, p-mTOR, p-BAD and Caspase-3 Expressions

**Figure 2:**
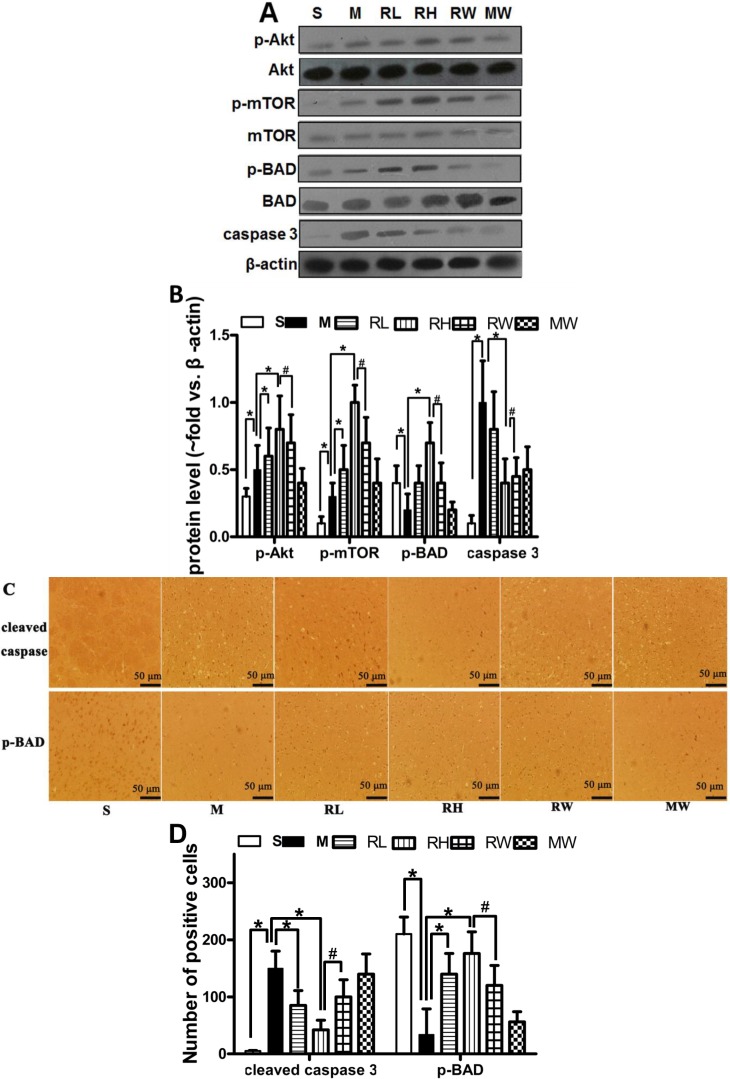
Effect of Rhy on the PI3K/Akt signaling and the downstream apoptosis-related proteins. (**A**) The expression of p-Akt, Akt, mTOR, p-mTOR, p-BAD, BAD, and cleaved caspase 3 in Sham (S), pMCAO (M), Rhy-L (RL), Rhy-H (RH), Rhy-H + wortmannin (RW) and pMCAO + wortmannin (MW) groups; (**B**) Densitometry analysis; (**C**) Immunohistochemical staining of p-BAD and cleaved caspase 3 at 24 h after pMCAO; (**D**) Bar graph shows quantitative data from each group (* *p* < 0.05 Rhy and Sham *vs.* pMCAO, ^#^ *p* < 0.05 RW *vs.* RH).

Western blotting analysis was used to estimate the activity of the PI3K/Akt signaling and the downstream apoptosis-related proteins (Figure 2A). In line with previous studies [18], up-regulated p-Akt and p-mTOR was observed in the pMCAO group compared to the Sham group. Compared to the pMCAO group and Sham groups, treatment with both high and low doses of Rhy considerably (*p* < 0.05) increased the ratio of p-Akt/Akt and p-mTOR/mTOR. Cerebral ischemia decreased p-BAD expression and increased cleaved caspase 3 expression as compared to the Sham group. Rhy-H preconditioning greatly (*p* < 0.05) prevented the p-BAD decrement and caspase-3 increment induced by the pMCAO. Wortmannin remarkably (*p* < 0.05) eliminated p-Akt, p-mTOR and p-BAD elevation induced by Rhy.

The immunohistochemical analysis confirmed the results obtained from western blotting. The immunopositive signals of cleaved caspase-3 in the infarct area were significantly elevated than the Sham group, whereas p-BAD expression was reduced (Figure 2C). Compared to the pMCAO group, Rhy treatment not only reduced the number of cells positive of cleaved caspse-3 but also increased that of p-BAD (*p* < 0.05). Treatment of wortmannin partially abolished the protective effects of Rhy-H.

### 2.3. Effect of Rhy on the Activation of TLRs/NF-κB Signaling

**Figure 3:**
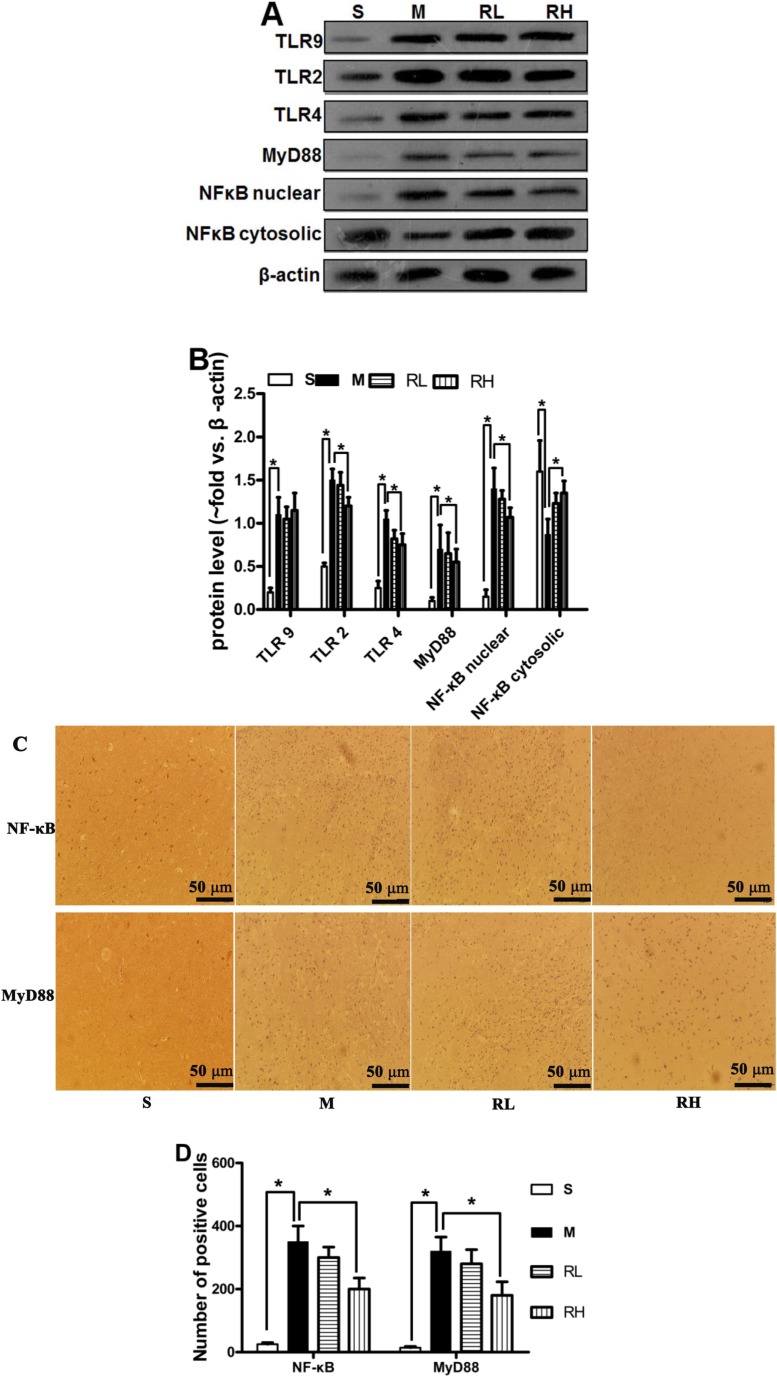
Effect of Rhy on the activation of TLR/NF-κB signaling. (**A**) The expression of TLR2/4/9, MyD88 and NF-κB at 24 h after pMCAO in Sham (S), pMCAO (M), Rhy-L (RL), Rhy-H (RH) groups. (**B**) Densitometry analysis. (**C**) Immunohistochemical staining of NF-κB and MyD88 in the cerebral cortex. (**D**) Bar graph shows quantitative data from each group (* *p* < 0.05 *vs.* pMCAO).

We analyzed the protein level of total TLR2, TLR4, TLR9, nuclear NF-κB and MyD88 with Western blot. In Sham group, the NF-κB was abundant in cytosolic fractions but scarce in nuclear extracts. While in pMCAO group, NF-κB level was expressively enlarged in nuclear fraction and poor in cytosolic fractions at 24 h after ischemia, signifying the translocation from the cytosol to the nucleus. TLR2, TLR4, TLR9 and MyD88 expressions were up-regulated 24 h post-pMCAO. Only high dose of Rhy was able to inhibit the expression of TLR2/4, MyD88 and NF-κB p65 translocation (*p* < 0.05, Figure 3A).

The localization of MyD88 and NF-κB was further determined by immunohistochemistry after stroke. Few cells positive of MyD88 and NF-κB were observed in the Sham group. Cerebral ischemia increased the cells positive of MyD88 and NF-κB than the Sham group. Consistent with western blotting results, high dose Rhy treatment considerably reduced expression of MyD88 and NF-κB (*p* < 0.05). Still, there were no significant differences between pMCAO and Rhy-L group (Figure 3C).

### 2.4. Effect of Rhy on the Expression of Claudin-5 and BDNF

**Figure 4:**
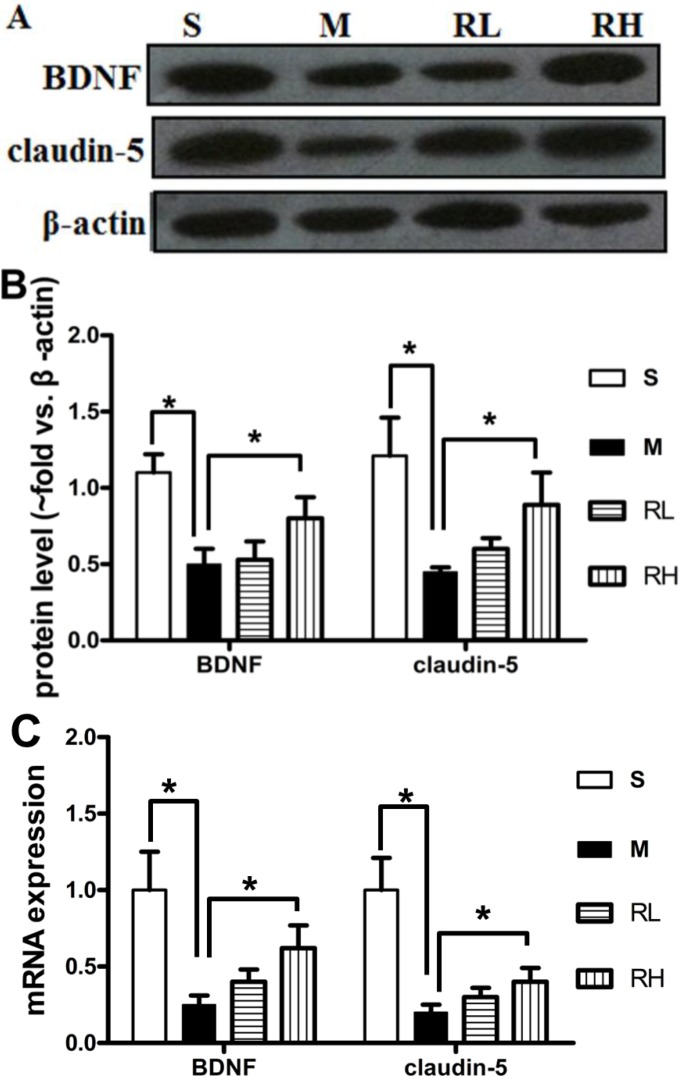
Effect of Rhy on the expression of claudin-5 and BDNF. (**A**). Expression of claudin-5 and BDNF at 24 h after pMCAO in Sham (S), pMCAO (M), Rhy-L (RL), Rhy-H (RH) groups. (**B**). Densitometry analysis. (**C**). The mRNA expressions of claudin-5 and BDNF were measured in Sham (S), pMCAO (M), Rhy-L (RL), Rhy-H (RH) groups (*p < 0.05 *vs.* pMCAO).

We further investigated the levels of the tight junction protein claudin-5 and brain-derived neurotrophic factor (BDNF), which could alleviate damage following ischemia [19,20]. In pMCAO group, claudin-5 was significantly declined than in the Sham group. Only Rhy-H could reverse claudin-5 decrease at both protein and mRNA levels (*p* < 0.05, Figure 4). The mean ratios of the BDNF densitometry data to those of β-actin in the Sham, pMCAO and Rhy-H groups were 0.55 ± 0.12, 0.31 ± 0.10, and 1.0 ± 0.14, respectively. The data above indicated the expression of BDNF was decreased after pMCAO surgery, while significantly upregulated after Rhy treatment. Similar results were obtained from RT-qPCR analysis (Figure 4C).

### 2.5. Discussion

pMCAO is a successful model to study cerebral ischemic damage in rats [21]. The cytotoxic responses, such as apoptosis, oxidative stress, proinflammation and neurological damage will initiate immediately upon the onset of cerebral ischemia. Anti-inflammation and anti-apoptosis therapies have attracted significant interest to combat ischemia-induced damage. Inhibiting inflammatory mediators displayed neuroprotection against ischemic brain damage [22]. Recent evidence suggests that TLRs may be the important targets for developing new treatment approaches for cerebral ischemia injury [23,24]. Accumulating evidence has demonstrated that TLR4 and TLR9 contribute to the inflammatory reaction [8,25]. Moreover, the expressions of TLR2 and TLR4 were enhanced after an ischemic stroke. Compared with wild-type mice, the neurological deficit and cerebral infarction induced by cerebral ischemia were remarkably attenuated in TLR2 or TLR4 knockout mice [26,27]. NF-κB, a key nuclear transcription factor, could combine with specific DNA sequence to trigger inflammation response and ischemic neuronal apoptosis [28]. TLRs were reported to induce NF-κB-associated pro-inflammatory cytokines through a MyD88-dependent pathway [29,30]. Cerebral ischemic injury activates TLR4-mediated signal transduction pathway and promote NF-κB translocation from the cytoplasm into nucleus. In our study, we observed the increment of TLR2, TLR4, nuclear NF-κB and MyD88 expressions at 24 h after ischemia, and high dose of Rhy considerably blunted the expression of these factors and inhibited p65 NF-κB transduction in ischemic brain. As an inflammatory mediator, TLR9 also involves in the process of ischemic damage [31], but Rhy fails to influence the expression of TLR9 in our study.

On the other hand, the PI3K/Akt pathway also renders neuroprotection after ischemic stroke [18]. Akt/mTOR signaling involves in cardiovascular disease and ischemic cardiomyocyte apoptosis [32,33]. In addition, Akt/mTOR signaling might relate to the protection against apoptosis in Parkinson’s and Alzheimer’s disease. Western blotting analysis revealed enhanced expression of p-mTOR in ischemia area in our study. The PI3K/Akt pathway promotes neovascularization and results in a noteworthy reduction in infarct size after ischemia [34]. However, there are inconsistent results about the level of phospho-Akt after ischemic injury. Osuka revealed that dysfunction of the Akt pathway was involved in ischemic damage [35], but Noshita and Shibata insisted that, within hours, pAkt transiently increase in neurons following cerebral ischemia [36,37], and this raise is regarded as a neuroprotective action. In Gao’s and Ishrat’s research, up-regulated pAkt was observed compared to sham group 24 h post-pMCAO [38,39]. Consistently, the same result was obtained in our manuscript.

Several reasons might contribute to the discrepancy in pAkt level. Firstly, p-Akt level depended on the timing after the injury/stress. After ischemia, pAkt was dephosphorylated immediately in the CA1 region and rephosphorylated after only 5 min of ischemia [40]. Secondly, the extent of ischemic injury also accounts-lethal damage downregulated pAkt in the CA1 region, but sublethal damage increased pAkt. Thirdly, the origin of tissue used in protein analysis also works. pAkt level was elevated in cortex in our manuscript or in peri-infarct cortical tissue in Ishrat’s research [39], or in the ipsilateral cerebral cortex as described by Pérez-Álvarez [41]. Both Gao and Shibata revealed that the enhanced pAkt occurred mainly in neurons located in the outer area of the middle cerebral artery territory (ischemic penumbra) [37,38], but Osuka reported that PI3K/Akt signaling was down-regulated in bilateral cortices adjacent to the hippocampus [35]. 

Cerebral ischemia motivates Akt and mTOR, which subsequently prevents BAD into the mitochondrial membrane, finally inhibiting death of neurons. Although BAD translocation into the mitochondrial membrane was inhibited by pAkt, phosphorylation of BAD was increased by activated Akt, thereby inhibiting the apoptotic activity and promoting cell survival [42]. As we know, p-BAD increment contributes to the inhibition of apoptosis. Apoptotic stimuli result in dephosphorylation of BAD, thus activating caspase-3 and Bax [43].

Our data show that Rhy reduced ischemic brain damage, probably by increasing the ratio of p-mTOR/mTOR, and triggering its downstream target p-BAD, thereby reducing ischemic neuronal death. These data resemble those reported by Ishrat that neuroprotective agents protect against stroke via mediating Akt/BAD phosphorylation [39,42]. Why there isn’t a direct and proportional relationship between pBAD and pAkt expression? It is reasonable that there is not enough pAkt to trigger pBAD at 24 h post-injury. Because the damage in the cortex region inflicted by the stroke model is relatively moderate. Herein, it is possible that Rhy treatment up-regulated pAkt in pMCAO rats and thereby increasing pBAD. Furthermore, in our study, wortmannin weakened the activation of BAD & Akt and thus abolished Rhy’s effects, confirming the critical role of PI3K/Akt pathway.

BDNF, which is widely distributed in central nervous system, protects tissue from injury and fosters neuronal plasticity via the PI3K/Akt pathway [18]. Akt pathway is an important downstream signaling pathway of BDNF. By interfering with apoptotic pathways, BDNF was able to protest cerebral ischemic injury [44,45]. In line with previous studies [46], pMCAO surgery resulted in a considerable shrink in BDNF level, which was then reversed by Rhy treatment. Consistently, Zhang and his co-workers demonstrated that the Akt/BDNF pathway could be activated to relieve ischemic injury [47].

## 3. Experimental Section

### 3.1. Animals

Male Sprague-Dawley rats (250–300 g), from the SLAC Lab Animal Center of Shanghai (Shanghai, China), were maintained on a standard diet and water *ad libitum* (light/dark cycle with humidity of 60% ± 5%, 22 ± 3 °C). The experimental procedures were approved by the Animal Ethics Committee of Jiangsu Provincial Academy of Chinese Medicine.

### 3.2. Surgery

As previously described, the pMCAO model was used to make permanent focal ischemia [48,49]. The left side of common carotid artery was isolated. Inserting a nylon mono-filament into the internal carotid artery about 18–20 mm until 80%–90% reduction of cerebral blood flow was observed by a blood flow monitor (Periflux system 5000, Perimed, Milan, Italy). Rectal temperature was kept at 36 °C to 38 °C in the surgery. The same surgical procedure was carried out in Sham group without inserting a filament. 

### 3.3. Experimental Design

Rhy (Zeji Medical Technology Co., Shanghai, China) with purity > 98% was dissolved in sterile physiological saline and intraperitoneally administrated once daily for four consecutive days before surgery and then received one more injection after surgery. Rats were randomly divided into 4 groups (20 rats in each group). Sham group; pMCAO group: pMCAO controls that received 0.9% saline; Rhy-L group: low-dose Rhy of 10 mg/kg; and Rhy-H group: high-dose Rhy of 30 mg/kg. Wortmannin (30 μg/kg) was given intravenously at 30 min post-pMCAO to explore the role of PI3K/Akt signaling in Rhy’s neuroprotective effects.

### 3.4. Neurobehavioral Evaluation

At 24 h after pMCAO surgery, the neurologic behaviors were evaluated as previously described [50]: (1) spontaneous activity; (2) symmetry in the movement of four limbs; (3) forepaw outstretching; (4) climbing; (5) body proprioception; (6) response to vibrissae touch. The score was the summation of all six individual test scores, from 3 to 18.

### 3.5. Infarct Volumes

After rats were sacrificed, the brains were removed quickly and coronally cut into 3-mm sections, and stained with 1% solution of 2,3,5-triphenyltetrazolium chloride (TTC) at 37 °C for 15 min. The infarcted brain seemes white, while the noninfarcted region closes to pink. The sections were photographed and measured with Image Pro-Plus 5.1 software. To eliminate the effect of brain edema, correction for edema of infarct area was achieved as previously described [51].

### 3.6. Brain Edema

Rats were killed 24 h after cerebral ischemia, and brain samples were obtained [52]. The ischemic area was carefully blotted with filter paper and weighed to get the wet weights (WW). Dry the ischemic hemispheres at 100 °C for 24 h, thus obtaining a constant weight as the dry weight (DW). Water content was calculated based on the formulation: H_2_O (%) = (WW − DW)/WW × 100%. 

### 3.7. Western Blot Analysis

The cortex tissues were lysed in lysis buffer, and the mixed liquor was centrifuged at 14,000 g for 5 min at the temperature of 4 °C. Nuclear protein extraction was carried out with the extraction kit (Keygen Biotech. Co., Ltd., Nanjing, China). Brain homogenate aliquots containing 50 μg protein were separated on 10% SDS-PAGE, transferred to polyvinylidene fluoride membranes (Millipore Corp., Billerica, MA, USA) and probed with antibodies included BAD, p-BAD, mTOR, p-mTOR, cleaved caspase 3, TLR2, TLR4, and TLR9 (diluted 1:500, Santa Cruz Biotechnology, Inc. Santa Cruz, CA, USA), Akt, p-Akt and Brain derived neurotrophic factor (BDNF) (1:1,000, Epitomics, Burlingame, CA, USA), NF-κB, myeloid differentiation factor 88 (MyD88) and claudin-5 (1:1,000, Proteintech, Chicago, IL, USA) and β-actin (1:4,000, Abcam, Cambridge, MA, USA). Protein bands were quantified and normalized to β-actin using Image J software. 

### 3.8. Immunohistochemical Rtudies

Sections (5 μm) from paraformaldehyde-fixed cortex tissues were dewaxed with xylene and graded ethanol series (100%, 95%, and 70%, v/v, 2 min each) and then washed in distilled water. The sections were incubated in 3% hydrogen peroxide to quench the activity of endogenous peroxidase. Sequentially, the slides were placed in citrate buffer and heated at 100 °C to retrieve antigens. The slides were incubated with MyD88 and NF-κB mouse polyclonal antibody (1:1,000, Proteintech), p-BAD and cleaved caspase-3 rabbit polyclonal antibodies (1:500, Santa Cruz) for 1 h, followed by incubation with horseradish peroxidase-conjugated secondary antibody. Sections were then washed with distilled water, incubated with diaminobenzidine-hydrogen peroxide, and counterstained with hematoxylin. Immunoreactivity was identified as brown nuclear counterstained with hematoxylin.

### 3.9. Real-Time Reverse Ranscription-Quantitative PCR (RT-qPCR)

Total RNA from ischemia cortex was isolated from the brain after pMCAO using TRIzol Reagent (Invitrogen, Carlsbad, CA, USA). Integrity of total RNA was assessed by agarose gel electrophoresis. BDNF: F: GGCTTGACATCATTGGCTGAC, R: CATTGGGCCGAACTTTCTGGT; Claudin-5: F: CTCTGCTGGTTCGCCAACAT, R: CAGCTCGTACTTCTGCGACA; GAPDH: F: AAGGTCGGT GTGAACGGATTT. R: AGATGATGACCCTTTTGGCCC. RT-PCR analysis was performed as follows: DNA was denatured at 94 °C for 3 min and cycled immediately for 35 cycles: denaturing at 94 °C for 45 s, annealing at 56 °C for 55 s, and extension at 72 °C for 1 min. The products were run on an agarose gel and stained with ethidium bromide and then photographed.

### 3.10. Statistics

The data are presented as the mean ± SE. The data from multiple groups were analyzed with one-way ANOVA and the Newman Keuls test for post hoc comparisons. Other data of the two groups were analysed using Student’s t-test. *p* values < 0.05 were regarded as statistically significant.

## 4. Conclusions

Our work suggests that the neuroprotective effects of Rhy are, at least partially, mediated via the activation of the PI3K/Akt pathway. In this experiment, we concentrated on the acute-stage treatment for ischemic damage. Further studies are warranted to investigate Rhy’s long-term effects.
